# Histopathological cutaneous alterations in systemic sclerosis: a clinicopathological study

**DOI:** 10.1186/ar3267

**Published:** 2011-02-28

**Authors:** Jens T Van Praet, Vanessa Smith, Marc Haspeslagh, Nele Degryse, Dirk Elewaut, Filip De Keyser

**Affiliations:** 1 Department of Rheumatology, Ghent University Hospital, De Pintelaan 185, BE-9000 Gent, Belgium; 2 Department of Dermatology, Ghent University Hospital, De Pintelaan 185, BE-9000 Gent, Belgium; 3 Department of Pathology, Stedelijk Ziekenhuis Roeselare, Brugsesteenweg 90, BE-8800 Roeselare, Belgium

## Abstract

**Introduction:**

The aims of the present study were to identify histopathological parameters which are linked to local clinical skin disease at two distinct anatomical sites in systemic sclerosis (SSc) patients with skin involvement (limited cutaneous systemic sclerosis (lcSSc) or diffuse cutaneous systemic sclerosis (dcSSc)) and to determine the sensitivity of SSc specific histological alterations, focusing on SSc patients without clinical skin involvement (limited SSc (lSSc)).

**Methods:**

Histopathological alterations were systematically scored in skin biopsies of 53 consecutive SSc patients (dorsal forearm and upper inner arm) and 18 controls (upper inner arm). Clinical skin involvement was evaluated using the modified Rodnan skin score. In patients with lcSSc or dcSSc, associations of histopathological parameters with local clinical skin involvement were determined by generalised estimation equation modelling.

**Results:**

The hyalinised collagen score, the myofibroblast score, the mean epidermal thickness, the mononuclear cellular infiltration and the frequency of focal exocytosis differed significantly between biopsies with and without local clinical skin involvement. Except for mononuclear cellular infiltration, all of the continuous parameters correlated with the local clinical skin score at the dorsal forearm. Parakeratosis, myofibroblasts and intima proliferation were present in a minority of the SSc biopsies, but not in controls. No differences were found between lSSc and controls.

**Conclusions:**

Several histopathological parameters are linked to local clinical skin disease. SSc-specific histological alterations have a low diagnostic sensitivity.

## Introduction

Systemic sclerosis (SSc) is a chronic autoimmune disease which can affect the skin and various internal organs [1]. One of the hallmark clinical features of SSc is skin thickening caused by oedema and excessive accumulation of collagen-rich extracellular matrix. Apart from sclerosis, the pathogenesis of SSc is characterized by vasculopathy, which is evidenced by nailfold capillary abnormalities and Raynaud's phenomenon, as well as by the presence of antinuclear antibodies such as anticentromere, anti-topoisomerase I and anti-RNA polymerase III antibodies. The most extensively validated technique to quantify skin involvement is the modified Rodnan skin score (mRSS) [2]. In this scoring system, 17 body areas are examined by clinical palpation and scored on the basis of judgement of skin thickness on a 4-point ordinal scale. Because the extent of skin disease correlates with the disease course, patients are grouped into disease subsets on the basis of skin involvement. The currently most widely applied classification differentiates three subsets, namely, limited SSc (lSSc), limited cutaneous SSc (lcSSc) and diffuse cutaneous SSc (dcSSc) [3]. Patients with lcSSc have skin involvement confined to the fingers, hands, forearms, lower legs or the face, whereas patients with dcSSc also have more proximal skin thickening. The group classified as lSSc has Raynaud's phenomenon with nailfold capillaroscopic abnormalities and/or SSc-associated autoantibodies, but no clinical skin involvement. This subset is considered to have 'early' SSc, as a longitudinal follow-up study has demonstrated that these patients are at risk for progression to definite SSc [4].

The histopathology of SSc skin has recently regained interest because it may be integrated as an outcome measure in clinical trials on skin disease, whereby skin biopsies are obtained before and after administration of a therapeutic drug [5,6]. In this way, the hyalinised collagen score and the myofibroblast score have previously been correlated with local skin score and durometry score in patients with dcSSc [7]. Consequently, these parameters could potentially be used to study the effect of a drug on skin disease. Other alterations in skin histology, however, have not been linked to clinical assessment. In addition, studies on the skin histopathology of SSc patients have mainly focused on alterations in patients with established disease, leaving open the question whether patients with 'early' disease have SSc specific histological alterations.

The aims of this study were (1) to identify histopathological parameters which are linked with local clinical skin disease at two different anatomical sites in SSc patients with skin involvement (lcSSc or dcSSc) and (2) to determine the sensitivity of SSc-specific histological alterations, with a focus on SSc patients with lSSc.

## Materials and methods

### Patients

Skin biopsies from the dorsal forearm (at the transition of the distal one-third and the proximal two-thirds) and the mid-upper inner arm were obtained from 53 consecutive SSc patients visiting the Scleroderma Clinic of the Ghent University Hospital. From two patients, only one biopsy was available for analysis. All patients fulfilled the criteria for early SSc set forth by LeRoy and Medsger [3]. Video nailfold capillaroscopy and antinuclear antibody identification were performed as described previously [8]. Patients were assigned to the lSSc, lcSSc or dcSSc group according to the criteria published by LeRoy *et al. *[9]. Skin involvement was clinically assessed according to the 17-site mRSS, whereby local skin involvement is determined for each site, including the dorsal forearm, on a semiquantitative scale (0 = normal thickness, 1 = mild thickening, 2 = moderate thickening and 3 = severe thickening) [10]. A normal reference set was included in the analysis. This set contained the skin biopsies from the inner upper arm of patients who were referred for a lupus band test and in whom further evaluation excluded any specified autoimmune disease. This study was conducted after approval of the Ghent University Hospital Ethical Committee was obtained and all patients had signed informed consent.

Full-thickness skin biopsies (approximately 1.5 cm long and 0.5 cm wide) were surgically obtained while the patients were under local anaesthesia. Biopsy samples were stored in formaldehyde and embedded in paraffin. Sections of 5 μm were cut and stained with haematoxylin and eosin, Masson trichrome and colloidal iron according to standard techniques. Paraffin-embedded sections were also used for immunohistochemistry.

### Immunohistochemistry

Paraffin-embedded sections were dewaxed and heated in antigen retrieval buffer at 98°C for 20 minutes using citrate buffer (pH 6) or Tris buffer (pH 9) (Pascal: Dako, Glostrup, Denmark). After rinsing and blocking endogenous peroxidase, sections were incubated for 60 minutes with the following mouse monoclonal antibodies (mAbs): CD3 (T cells; Novocastra, Newcastle, UK) and α-smooth muscle actin (α-SMA) (myofibroblasts, clone 1A4; Dako). Parallel sections were incubated with irrelevant isotype-matched mAbs as negative controls. The sections were subsequently incubated for 15 minutes with a biotinylated antimouse secondary antibody, followed by 15-minute incubation with a streptavidin-peroxidase complex (LSAB+ Kit; Dako). The colour reaction was developed using 3-amino-9-ethylcarbazole substrate (Dako) as chromogen. Finally, the sections were counterstained with haematoxylin. All incubations were carried out at room temperature, and the sections were washed with phosphate-buffered saline between all steps.

### Selection of scoring parameters for SSc histological skin alterations

Studies of SSc skin histopathology have described alterations in different skin compartments, including atrophy and increased pigmentation of the epidermis [11], loss of the epidermal papillae [11], increase of melanophages ('pigment incontinence') [11], the presence of a mononuclear perivascular infiltrate and myofibroblasts [7,12-14], sclerosis [7], narrowing of arteriolar lumina in the deep vascular plexus (reticular dermis) [15] and disappearance and entrapment of dermal adnexae and calcification [11,12]. To this set of alterations, we added two key histopathological features of other scleroderma-like disorders, namely, mucin deposition and fibroplasia [16,17]. Because analysis of routine biopsies revealed the presence of telangiectasia, focal exocytosis (that is, the presence of lymphocytes in the epidermis) and parakeratosis in some SSc biopsies, these items were also added to the set of scoring parameters [18]. Scoring systems for different parameters were obtained from previous publications as much as possible. An overview of the skin parameters that were scored is shown in Table 1.

**Table 1:** Overview of the skin histology parameters and scoring system^a^

Method	Parameter	Scoring system
Histology (H&E)	Epidermal pigmentation	10-cm VAS
Histology (MT)	Hyalinised collagen	10-cm VAS [7]
IHC (α-SMA)	Myofibroblasts	10-cm VAS [7]
Histology (H&E)	Mean epidermal thickness	Semiquantitative
Histology (H&E)	Mononuclear cellular infiltration	Semiquantitative [14]
Histology (CI)	Mucin deposition in deep dermis	Semiquantitative [16]
Histology (H&E)	Calcification	Present or absent
Histology (MT)	Entrapment of an eccrine sweat gland	Present or absent
Histology (H&E)	Fibroplasia	Present or absent
IHC (CD3)	Focal exocytosis	Present or absent
Histology (H&E)	Loss of adnexae	Present or absent
Histology (H&E)	Loss of epidermal papillae	Present or absent
Histology (H&E)	Intima proliferation of deep arterioles	Present or absent
Histology (H&E)	Parakeratosis	Present or absent
Histology (H&E)	Pigment incontinence	Present or absent
Histology (H&E)	Telangiectasia in papillary dermis	Present or absent

^a^α-SMA, α-smooth muscle actin; CI, colloidal iron; H&E, haematoxylin and eosin; IHC, immunohistochemistry; MT, Masson trichrome; VAS, Visual Analogue Scale.

**Figure 1:**
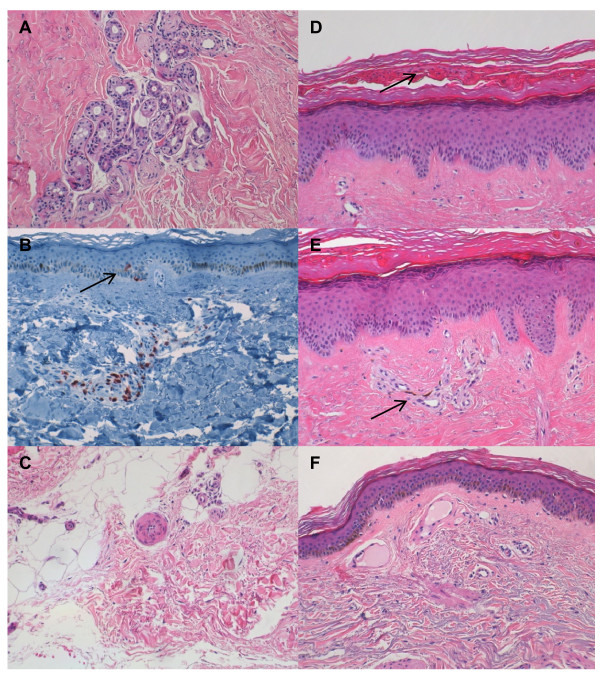
**Histopathological alterations in the skin of SSc patients**. All photographs were taken at ×200 magnification. **(A) **Entrapment of an eccrine sweat gland. **(B) **Focal exocytosis (arrow). **(C) **Intima proliferation in a deep arteriole. **(D) **Parakeratosis (arrow). **(E) **Pigment incontinence (arrow). **(F) **Telangiectasia. **(A **and **C-F) **Haematoxylin and eosin staining. **(B) **Anti-CD3 staining.

The hyalinised collagen score and the myofibroblast score were assessed on a 10-cm Visual Analogue Scale [5]. The epidermal thickness was scored using a semiquantitative scale on the basis of the mean number of keratinocyte layers (stratum basale and stratum spinosum) in a zone between two rete ridges (that is, undulations of the dermoepidermal junction) in five microscopic fields chosen at random (0 = mean less than three layers, 1 = mean of three or four layers, 2 = mean of five or six layers, 3 = mean of more than six layers), a scoring system which has also been used for the synovial lining layer [14,19]. Mononuclear cellular infiltration was scored on a semiquantitative scale as 0 (few scattered cells), 1 (maximum number of cells per collection at least 10), 2 (maximum number of cells per collection between 10 and 50) or 3 (maximum number of cells per collection at least 50) [14]. Mucin deposition in the reticular dermis was evaluated by scoring the degree of acid mucopolysaccharide staining on a semiquantitative scale as negative, very slight, slight, fair or abundant [16]. Entrapment of an eccrine sweat gland was defined as the absence of any surrounding fat tissue (Figure 1A). Focal exocytosis was defined as the presence of T-lymphocytes in the epidermis at least at two distinct sites (Figure 1B). Intima proliferation in deep arterioles was considered pathologic if the vessel wall thickness exceeded the diameter of the vessel lumen (Figure 1C). Parakeratosis was defined as the presence of nuclei in the stratum corneum (Figure 1D). Pigment incontinence was defined as the presence of melanin in papillary macrophages at least at two distinct sites (Figure 1E). Telangiectasia was defined as enlarged papillary capillaries (Figure 1F).

Stained slides were coded so that a blinded analysis could be performed. Slides from the dorsal forearm were analysed by two independent observers (MH and JTVP) who were uninformed of any clinical data. All scoring parameters were judged to be reliable, as the interobserver agreement was substantial (κ > 0.6 for all categorical parameters and intraclass correlation coefficient >0.7 for all continuous parameters). For continuous parameters, the mean of the two observers was used for analysis. In case of a discrepant score for a categorical parameter, a consensus was determined by the two observers. Slides from the upper inner arm were scored twice by one observer (JTVP). For continuous parameters, the mean of the two scores was used for analysis. In case of a discrepant score for a categorical parameter, a consensus was determined by a third evaluation. Because fibroplasia and calcification were not observed in a single biopsy, these items were left out of all analyses.

### Statistical analysis

In patients with lcSSc or dcSSc, associations of histopathological parameters with local clinical skin involvement were determined by generalised estimation equation (GEE) modelling with a correction for subject level and biopsy site. Local skin involvement at the dorsal forearm was defined as a local clinical score of at least 1. Since the upper inner arm local skin score is not included in the mRSS, all patients with dcSSc were considered to have local skin involvement at this site. In case a significant interaction between biopsy site and local clinical skin involvement was found in the GEE model, statistical testing for the effect of skin involvement was separately performed for the dorsal forearm and the upper inner arm. For normally distributed parameters, the Pearson correlation coefficient was used to determine the correlation with the dorsal forearm score. Otherwise, the Spearman correlation coefficient was used. For categorical parameters, Fisher's exact test was used to analyse the association with the dorsal forearm score. Mann-Whitney *U *test (continuous data) and Fisher's exact test (categorical data) were used to compare lSSc biopsies with normal controls. *P *≤ 0.05 was considered statistically significant. All analyses were performed using PASW 18.0 software (SPSS, Inc., Chicago, IL, USA).

## Results

### Clinical characteristics of the patients

Skin biopsies from the dorsal forearm and the upper inner arm were obtained from 53 consecutive SSc patients (17 males and 36 females; mean age ± SD, 52 ± 12 years). Seven SSc patients (13%) had no clinical skin involvement (lSSc), and 46 SSc patients (87%) had skin involvement (29 lcSSc and 17 dcSSc patients). Twenty-five patients used methotrexate, and 11 patients used low-dose corticosteroids (< 15 mg prednisolone/day). Table 2 summarizes the features of the different SSc patient subsets. Normal skin samples taken from the upper inner arm were included as a reference set (*n *= 18 comprising 7 males and 11 females; mean age ± SD, 44 ± 17 years).

**Table 2:** Clinical data of patients with lSSc, lcSSc and dcSSc^a^

Subset	lSSc (*n *= 7)	lcSSc (*n *= 29)	dcSSc (*n *= 17)
Mean age, yr (± SD)	51 ± 16	51 ± 13	55 ± 8,0
Female/male, *n*	6/1	21/8	9/8
Median disease duration^b ^(range^c^)	2 (2 to 21)	6 (0 to 35)	2 (0 to 12)
Median mRSS (range)	0 (0 to 0)	4 (0 to 14)	21 (4 to 27)
Median local skin score dorsal forearm (range)	0 (0 to 0)	0 (0 to 2)	1 (0 to 3)
ACR criteria, *n*	0	14	17
ANA, *n*			
Topoisomerase I	0	5	8^c^
Centromere	6	14	3^c^
RNA polymerase III	0	1	3
U1-RNP	0	3	0

^a^ACR, American College of Rheumatology; ANA, antinuclear antibodies; dcSSc, diffuse cutaneous systemic sclerosis; lSSc, limited systemic sclerosis; lcSSc, limited cutaneous systemic sclerosis; mRSS, modified Rodnan skin score; U1RNP, U1-ribonucleic protein; ^b^disease duration from first non-Raynaud's phenomenon symptom; ^c^one patient had both anticentromere and anti-topoisomerase I antibodies. ^c^range denotes the full interval between the smallest and largest values

### Associations of local clinical skin involvement and histological alterations

To examine associations of local skin disease with histological parameters, we analysed the biopsies from patients with lcSSc or dcSSc (*n *= 46). We found that, independently of the anatomical site of the biopsy, the hyalinised collagen score, the myofibroblast score, the mean epidermal thickness, the mononuclear cellular infiltration and the frequency of focal exocytosis differed significantly between biopsies with and without local skin involvement (α = 0.05; GEE) (Table 3). For the continuous parameters, only the epidermal thickness (*r *= 0.553; *P *< 0.001), the myofibroblast score (*r *= 0.507; *P *< 0.001) and the hyalinised collagen score (*r *= 0.572; *P *< 0.001) correlated with the local clinical skin score. No association was found between the local clinical score and the frequency of focal exocytosis (*P *= 0.06).

**Table 3:** Histological characteristics of patients with lcSSc or dcSSc^a^

	Dorsal forearm		Upper inner arm		
Histological characteristics	No local skin involvement (*n *= 24)	Local skin involvement (*n *= 22)	No local skin involvement (*n *= 29)	Local skin involvement (*n *= 16)	*P *value^b^
Mean epidermal pigmentation (± SD)	33 ± 22	37 ± 23	17 ± 18	40 ± 20	NR^c^
Mean hyalinised collagen (± SD)	20 ± 21	42 ± 29	11 ± 13	43 ± 33	< 0.001
Mean myofibroblast score (± SD)	1.1 ± 4.9	12 ± 21	0.79 ± 3.8	14 ± 27	0.004
Mean epidermal thickness (± SD)	1.6 ± 0.58	2.3 ± 0.61	1.1 ± 0.51	1.5 ± 0.48	< 0.001
Mean mononuclear cellular infiltration (± SD)	0.89 ± 0.34	1.1 ± 0.38	1.4 ± 0.79	1.9 ± 0.77	0.005
Mean mucin deposition in deep dermis (± SD)	1.7 ± 1.3	2.1 ± 1.4	0.96 ± 0.83	1.5 ± 1.2	0.09
Entrapment of eccrine sweat gland, *n *(%)	10 (42)	11/21 (52)	8 (28)	6/15 (40)	0.284
Focal exocytosis, *n *(%)	1 (4.2)	5 (23)	14 (48)	11 (69)	0.043
Loss of adnexae, *n *(%)	10 (42)	12 (55)	9 (31)	3/15 (20)	0.94
Loss of epidermal papillae, *n *(%)	10 (42)	5 (23)	1 (3,4)	0 (0)	0.122
Intima proliferation of deep arterioles, *n *(%)	3 (13)	6 (27)	5 (17)	2/15 (13)	0.596
Parakeratosis, *n *(%)	0 (0)	3 (14)	0 (0)	0 (0)	NR^d^
Pigment incontinence, *n* (%)	9 (38)	13 (59)	16 (55)	9 (56)	0.141
Telangiectasia in papillary dermis, *n *(%)	2 (8,3)	0 (0)	0 (0)	0 (0)	NR^d^

^a^lcSSc, limited cutaneous systemic sclerosis; dcSSc, diffuse cutaneous systemic sclerosis; SD, standard deviation; NR, not reported; ^b ^generalised estimation equation (GEE) modelling was used to determine the effect of local skin involvement for each variable, correcting for subject level and biopsy site; ^c^in the GEE model, the effect of this parameter differed significantly between the skin sites (Student's *t*-test was used to evaluate the upper inner arm, *P *< 0.001, and the dorsal forearm, *P *= 0.580); ^d^statistical testing could not be performed.

### Sensitivity and specificity of histological alterations

To determine the specificity of the studied histological parameters, we analysed 18 upper inner arm biopsies from patients who were referred for a lupus band test and in whom further evaluation excluded any specified autoimmune disease. Myofibroblasts, intima proliferation of deep arterioles and parakeratosis were not seen in these biopsies (Table 4). Comparison with the biopsies of the patients with lSSc (*n *= 7) revealed no statistically significant differences (Table 4). Analysis of all SSc biopsies showed that myofibroblasts, intima proliferation of deep arterioles and parakeratosis were present in, respectively, 22%, 19% and 5.8% of the dorsal forearm biopsies and in 14%, 14% and 0% of the upper inner arm biopsies.

**Table 4:** Histological characteristics of patients with lSSc and controls^a^

	Upper inner arm		
Histological characteristics	lSSc (*n *= 7)	Controls (*n *= 18)	*P *value
Mean epidermal pigmentation (± SD)	22 ± 23	16 ± 20	0.532
Mean hyalinised collagen (± SD)	2.3 ± 2.4	2.1 ± 1.9	0.893
Myofibroblast score (± SD)	0 ± 0	0 ± 0	NR^b^
Mean epidermal thickness (± SD)	0.86 ± 0.48	0.92 ± 0.31	0.714
Mononuclear cellular infiltration (± SD)	1.3 ± 1.1	1.5 ± 0.8	0.650
Mucine deposition in deep dermis (± SD)	1.2 ± 0.49	1.3 ± 0.7	0.663
Entrapment of eccrine sweat gland, *n *(%)	3 (43)	1/13 (7.7)	0.101
Focal exocytosis, *n *(%)	2 (29)	5/17 (16)	1.0
Loss of adnexae, *n *(%)	1 (14)	7 (50)	0.174
Loss of epidermal papillae, *n *(%)	2 (29)	2 (11)	0.548
Intima proliferation of deep arterioles, *n *(%)	0 (0)	0 (0)	NR^b^
Parakeratosis, *n *(%)	0 (0)	0 (0)	NR^b^
Pigment incontinence, *n *(%)	1 (14)	3 (17)	1.0
Telangiectasia in papillary dermis, *n *(%)	1 (14)	1 (5.6)	0.490

^a^lSSc, limited systemic sclerosis; NR, not reported; ^b^statistical testing could not be performed.

## Discussion

Because of the rarity of the disease, few studies have systemically addressed the skin histopathology of SSc. Previous studies may have been hampered by bias due to the inclusion of only dcSSc patients [7], by failure to perform biopsies at the same anatomical site in all patients [15] or by failure to include a normal control group or to link alterations to clinical scoring [11,13]. The present study was designed to overcome these issues and to identify histopathological alterations in the skin of SSc patients which are linked to clinical skin scoring or might have diagnostic relevance.

The results of this study show that independent of the biopsy site (dorsal forearm or inner upper arm), the hyalinised collagen score, the myofibroblast score and the mean epidermal thickness are associated with the presence of local clinical skin involvement. Furthermore, these three parameters correlated well with the local clinical skin score at the dorsal forearm. In agreement with our data, Kissin *et al. *[7] reported a good correlation of the hyalinised collagen score and the myofibroblast score with clinical scoring in patients with dcSSc. The link between histopathological alterations and clinical assessment at two independent skin sites in a large set of SSc biopsies, including patients from different disease subsets and with early and late disease, suggests these parameters might be potential candidates as outcome measures in clinical trials on skin disease. However, longitudinal studies should address their sensitivity to change before they can be considered validated measures [20]. Given that the link with clinical scoring was independent of the skin site, our data indicate that in patients with dcSSc, biopsies from the upper inner arm may be used to study histological parameters.

One interesting finding of our study is the link between clinical skin scoring and epidermal changes. Apart from the increased mean epidermal thickness in biopsies with local clinical skin involvement, we also found parakeratosis in a minority of clinically involved skin biopsies from the dorsal forearm, which points to a disturbance of epidermal differentiation [21]. Consistent with these results, Aden *et al. *[22] demonstrated that the epidermis in involved SSc skin shows thickening and altered differentiation, mimicking an active wound-healing phenotype. In contrast, older literature reported atrophy of the epidermis in SSc skin biopsies [11]. Also, we found that local clinical skin involvement was associated with a higher epidermal pigmentation at the upper inner arm but not at the dorsal forearm, which is probably related to the different sun exposure of the two biopsy sites.

A second research aim of this study was to determine the sensitivity of SSc-specific histological alterations, focusing on SSc patients without clinical skin involvement (lSSc). These patients have Raynaud's phenomenon and SSc-associated antinuclear antibodies and/or nailfold capillaroscopic alterations without skin involvement. At the upper inner arm, we found no differences between lSSc and control biopsies. Concerning the specificity of histological parameters for SSc, we could not detect parakeratosis, myofibroblasts or intima proliferation of the deep arterioles in controls. However, these alterations were present in only a minority of the SSc biopsies. Thus, SSc-specific histological alterations have a low diagnostic sensitivity.

## Conclusions

In conclusion, the systematic analysis of skin biopsies from 53 consecutive SSc patients and 18 normal controls revealed that the mean epidermal thickness, the hyalinised collagen score and the myofibroblast score are linked to local clinical skin involvement and are correlated with the local skin score. Concerning histological alterations in lSSc, we found no significant differences with control skin at the upper inner arm. Finally, myofibroblasts, intima proliferation of the deep arterioles or parakeratosis in a skin biopsy are useful diagnostic markers for SSc, although they have a low sensitivity.

## Abbreviations

dcSSc: diffuse cutaneous systemic sclerosis; GEE: generalised estimation equation; lcSSc: limited cutaneous systemic sclerosis; lSSc: limited systemic sclerosis; mRSS: modified Rodnan Skin Score; SSc: systemic sclerosis.

## Competing interests

The authors declare that they have no competing interests.

## Authors' contributions

JTVP, VS, MH and FDK designed the study. VS acquired the capillaroscopic and clinical data. JTVP and FDK acquired the serological data. JTVP, MH and ND acquired the histological data. JTVP, VS, MH, DE and FDK participated in the manuscript preparation and finalisation. All authors read and approved the final manuscript.
